# High prevalence of dengue, Zika, and chikungunya viruses in blood donors during a dengue outbreak and an endemic period in Colombia

**DOI:** 10.3389/fmed.2024.1380129

**Published:** 2024-05-01

**Authors:** Brian Alejandro Cáceres Munar, Adriana Urbina, Tatiana Ortíz, Ayda Rodríguez, Olga Lucía Fernández, Luisa Fernanda Ospina, Iris Flórez, Dora Uribe, Celia Alvarado, Eliana Patricia Calvo, Félix Giovanni Delgado, Jaime Eduardo Castellanos

**Affiliations:** ^1^Grupo de Virología, Vicerrectoría de Investigaciones, Universidad El Bosque, Bogotá, Colombia; ^2^Fundación Universitaria Sanitas, Bogotá, Colombia; ^3^Banco Nacional de Sangre Cruz Roja Colombiana, Bogotá, Colombia; ^4^Banco de Sangre Quindío, Cruz Roja Colombiana, Armenia, Colombia; ^5^Hemocentro Valle del Cauca, Cruz Roja Colombiana, Cali, Colombia; ^6^Banco de Sangre Bolívar, Cruz Roja Colombiana, Cartagena, Colombia; ^7^Hemocentro del Café, Cruz Roja Colombiana, Manizales, Colombia; ^8^Banco de Sangre Antioquia, Cruz Roja Colombiana, Medellín, Colombia

**Keywords:** dengue virus, Zika virus, chikungunya virus, blood donors, blood components, blood transfusion, blood banks, Colombia

## Abstract

**Objective:**

Arboviruses pose a challenge in ensuring the supply of pathogen-free blood components because they are not routinely screened in blood banks, and blood components from infected asymptomatic donors could be transfused. This study aimed to detect and characterize arboviral infections in Colombian blood donors.

**Methods:**

In a cross-sectional study, the prevalence of dengue (DENV), Zika (ZIKV), and chikungunya (CHIKV) viruses and co-infections of blood donors were compared between an epidemic period (November 2019–February 2020, *n* = 462) and an endemic period (November 2021–August 2022, *n* = 1,119). Viral RNA from each donor serum was purified, and the viruses were detected using a previously standardized multiplex hemi-nested RT-PCR protocol. Subsequently, donors who tested positive were surveyed 15 days after the detection of the virus to identify clinical characteristics related to the arboviral infection. The prevalences of each virus were presented as percentages and compared between epidemic and endemic periods.

**Results:**

Significantly higher prevalences were found in the epidemic period compared with the endemic period for DENV (14.5 vs. 1.9%), ZIKV (7.8 vs. 0.3%), CHIKV (8 vs. 3.3%), and co-infections (4.3 vs. 0.2%). The survey response rate of positive donors in the two periods was 83/175 (47%). In total, 57% of the donors surveyed were asymptomatic. Symptomatic donors most frequently reported headache (31%), malaise (13%), arthralgia (10%), and fever/chills (8%).

**Conclusion:**

The prevalence observed in epidemic and endemic periods was higher than that reported in other studies in the Americas. The high proportion of asymptomatic cases found, in addition to the mild and nonspecific manifestations among the symptomatic, may limit the effectiveness of the donor selection criteria used to mitigate the risk of transfusion-transmitted arboviruses.

## 1 Introduction

Dengue (DENV), Zika (ZIKV), and chikungunya (CHIKV) are positive-strand RNA viruses (1–3) that cause a broad spectrum of clinical manifestations in the host (4–6). These viruses are classified as arboviruses and are generally transmitted through the bite of infected female *Aedes* mosquitoes. However, transfusion-transmitted infection (TTI) has recently been established as an alternative transmission route for these viruses, posing potential public health risks (7–10). Key factors such as the high rate of subclinical infections (11–13) and the high viral loads detected in infected blood donors support this assertion (14–20).

Since 2009, the Association for the Advancement of Blood and Biotherapies (AABB) has listed emerging infectious agents and classified them based on their potential risk for blood safety (9). According to the AABB, DENV has the highest priority level (red) due of to its potentially severe clinical outcomes when transmitted through blood transfusion. CHIKV is also included but at a lower priority level (orange). In addition, ZIKV was included (21) because its active transmission has been reported in 58 countries, and probable transfusion transmission cases have been disclosed in French Polynesia and Brazil (22–24).

Over the last 20 years, a significative circulation of arbovirus in blood donors has been documented during epidemics, with 5.5% of donors positive for DENV RNA in Saudi Arabia (25), 2.8% positive for ZIKV RNA in French Polynesia (26), and 1.9% positive for CHIKV in Puerto Rico (20). Moreover, infectious DENV and transmission cases have been associated with plasma, red blood cells, and platelet transfusions (27, 28). In contrast, transfusion transmission of ZIKV has only been observed during platelet transfusion (23). Although transfusion-transmitted cases of CHIKV infection have not been reported yet, it is considered a high-risk virus for future TTIs because of the high levels of viremia in asymptomatic infected donors (29).

Colombia is a hyperendemic country in which all four DENV serotypes, CHIKV, and ZIKV (30) circulate, and the *Aedes aegypti* vector is present in more than 90% of the territory (30). Therefore, it is estimated that more than half of the Colombian population is at risk of being infected by these arboviruses (31), especially during outbreaks that occur during periods of drought and high temperatures (32). To date, five dengue outbreaks (1998, 2002, 2010, 2013, and 2019), one CHIKV outbreak (2016), and one ZIKV outbreak (2015) have been reported in the country (33–35). Indeed, during the most recent DENV outbreak in 2019, 124,989 cases were reported, of which 48.1% had dengue without warning signs, 50.8% presented dengue without warning signs, and 1.1% had severe dengue (36). Hence, dengue fever is the most prevalent vector-borne viral disease in the country, with more than 1,500,000 cases registered from 1990 to 2016 (31), followed by CHIKV with over 460,000 cases (37–39) and ZIKV with over 100,000 confirmed cases (40).

Although no cases of arbovirus transmission by blood transfusion have been reported yet in Colombia, information should be collected to inform future public health policies. This study aimed to identify and characterize DENV, ZIKV, and CHIKV infections in blood donors residing in endemic and nonendemic cities who visited blood banks within the Colombian Red Cross network during the most recent dengue outbreak and a subsequent DENV endemic period. Our results showed a high prevalence of all these viruses, although DENV and CHIKV were the most frequently reported viruses during both epidemiological periods. Moreover, a clinical and epidemiological survey of the infected blood donors showed that many cases were asymptomatic and that among the symptomatic cases, the clinical manifestations were mild and nonspecific.

## 2 Materials and methods

### 2.1 Serum samples

A total of 1,581 serum samples from accepted blood donors were collected from six blood banks across Colombia in hyperendemic cities located below 1,800 m above sea level (m.a.s.l.) (Armenia, Cali, Cartagena, and Medellín) and those located above 1,800 m.a.s.l. (Bogotá and Manizales). Each blood bank collects blood from these cities and their nearby municipalities. Acceptability for blood donation was determined according to the National Technical Guideline for the Selection of Blood Donors (41). The blood donors provided informed consent for blood donation and collection of an additional blood sample to detect arboviruses. The Universidad El Bosque Institutional Ethics Committee approved this study.

During blood donation, 7 mL of blood were collected using a yellow cap tube to obtain serum. Sera from blood donors were stored in each blood bank at a temperature between −20 and − 30°C and sent to the Instituto de Virología at the Universidad El Bosque in the capital city, Bogotá, for processing within 7 days after collection.

Sera were sampled during two different periods: First, during a dengue outbreak from November 2019 to February 2020 (*n* = 462); second, during a dengue endemic period from November 2021 to August 2022 (*n* = 1,119; Table 1). Samples were classified as coming from an endemic or nonendemic area for arboviruses, according to the altitude above sea level of the donation site. In Colombia, it has been shown that the *Aedes aegypti* vector infected with at least one arbovirus circulates up to 1,984 m.a.s.l. (42). Therefore, samples collected between 0 and 1,984 m.a.s.l. were classified as coming from arbovirus-endemic areas, whereas those collected at higher altitudes were classified as coming from nonendemic areas.

**Table 1:** Origin and distribution of serum samples from accepted blood donors for arboviral detection.

Blood banks	Dengue outbreak 2019–2020		Dengue endemic phase 2021–2022	
	No. samples	Percentage	No. samples	Percentage
Armenia (Quindío)	26	5.6	42	3.8
Cali (Valle)	87	18.8	261	23.3
Cartagena (Bolívar)	125	27.1	235	21.0
Medellín (Antioquia)	17	3.7	86	7.7
Bogotá (Bogotá D.C.)	130	28.1	256	22.9
Manizales (Caldas)	77	16.7	239	21.4
Total	462	100.0	1,119	100.0

City (Province).

### 2.2 One-step hemi-nested RT-PCR for DENV, ZIKV, and CHIKV simultaneous detection

A volume of 200 μL of serum was used to purify RNA using a commercial DNA/RNA Virus Mini Kit (Stratec). Culture supernatants (200 μL) from DENV-infected C6/36 cells (DENV-1 S24, DENV-2 S3, DENV-3 S7, and DENV-4 S29 strains) and ZIKV (COL8565 strain)- or CHIKV (COL7624 strain)-infected Vero cells were used as positive controls. These viral isolates had undergone an adaptation process involving 4–5 passages. Purified arboviral RNA was amplified using a one-step multiplex hemi-nested RT-PCR (hnRT-PCR) previously described by our group (43). Briefly, the first round of amplification was performed using a final volume of 20 μL using Luna Universal Probe One-Step RT-qPCR (NEB), 5 μL of template RNA (60–80 ng/μL), and 0.2 μM of each primer (a total of six primers). The amplification temperatures were 15 min at 55°C and 3 min at 95°C; 30 s at 95°C, 30 s at 55°C, and 30 s at 72°C, for 30 amplification cycles; and finally, 5 min at 72°C.

**Figure 1:**
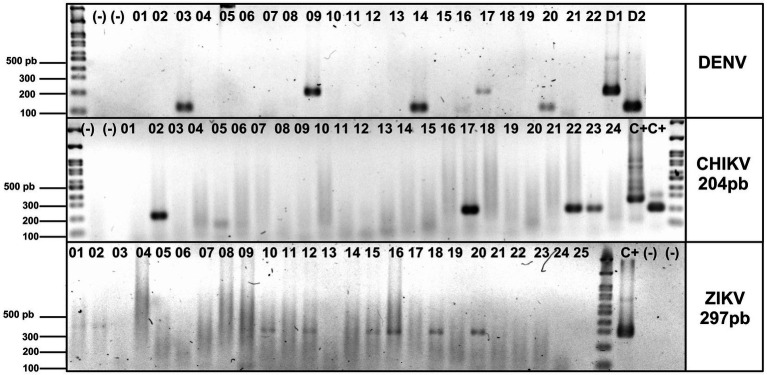
Detection of DENV, ZIKV, and CHIKV in blood donor sera by multiplex semi-nested RT-PCR. Specific DENV, ZIKV, and CHIKV products were separated on a 2% agarose gel and stained with ethidium bromide, a DNA molecular weight marker (100 bp ladder), to verify amplicon size. Blood donor sera are between lanes 01 and 26, negative controls: (−), positive controls: D1, D2, and C+. C+* plasmid DNA used as internal laboratory control.

The four DENV serotypes, ZIKV, and CHIKV were detected in separate reactions in a final volume of 20 μL, using 2 μL of template (from the first round of amplification), 3 mM MgCl_2_, 0.4 mM dNTPs, and 0.2 μM of each specific primer. The amplification program was as follows: 3 min at 95°C; 30 s at 95°C, 30 s at 55°C (ZIKV and DENV) or 30 s at 60°C (CHIKV); 30 s at 72°C for 30 cycles of amplification and 5 min at 72°C. The products were visualized using ethidium bromide on 2% agarose gels. Figure 1 shows representative samples for arbovirus RNA detection.

In addition, the analytical sensitivity of the hnRT-PCR assay was determined by testing viral RNA obtained from serial dilutions of laboratory virus strains that had been previously titrated. The limit of detection (LoD) for DENV-2, ZIKV, and CHIKV was 1 × 10^2^, 6 × 10^0^, and 1 × 10^2^ viral RNA copies/mL, respectively. Similarly, an alternative RT-qPCR assay to detect these viruses was evaluated under the same test conditions, resulting in LoDs of 1 × 10^3^, 6 × 10^0^, and 4 × 10^4^ viral RNA copies/mL, respectively. Consequently, the hnRT-PCR assay exhibited a higher analytical sensitivity compared to the RT-qPCR assay.

### 2.3 Survey of blood donors positive for arbovirus

Two weeks after blood donation, a healthcare professional contacted the blood donors who tested positive for at least one of the tested arboviruses to record their sociodemographic, clinical, and epidemiological characteristics.

### 2.4 Statistical analysis

The positivity rates for arboviruses are presented as prevalences (percentages) and their 95% confidence intervals (95% CI). Categorical variables are presented as frequencies. Comparisons between the dengue outbreak and the endemic period and between arbovirus-endemic and nonendemic areas were performed using the chi-square or Fisher’s exact test. The significance level was set to *p* < 0.05. Statistical analyses were performed using Stata v16.

## 3 Results

### 3.1 Prevalence of arboviral RNA in blood donors in Colombia

We collected 1,581 samples from six blood banks in Colombia between 2019 and 2022. After individual sample screening, arboviral RNA was detected in 175 of the 1,581 samples tested, indicating a prevalence of arboviral RNA of 11% in accepted blood donors. The prevalence of DENV was 88/1,581 (5.6%), CHIKV was 74/1,581 (4.7%), and ZIKV was 39/1,581 (2.5%). Interestingly, in addition to single-virus infections, we found co-infections in 23/1,581 donors (1.4%).

### 3.2 The prevalence of arboviral RNA in blood donors changed between the outbreak and endemic periods

Considering that samples were collected in two different epidemiological periods, we calculated the prevalence of arboviral RNA separately for each one: the dengue outbreak in 2019–2020 (*n* = 462) and an endemic period in 2021–2022 (*n* = 1,119). As shown in Table 2, arboviral RNA from DENV, ZIKV, CHIKV, and co-infections were detected in blood donors, although their prevalences significantly differed between these two epidemiological periods.

**Table 2:** Prevalence of arboviral RNA in blood donors in Colombia during a dengue outbreak (2019–2020) and an endemic period (2021–2022).

Variable	Dengue outbreak 2019–2020			Dengue endemic phase 2021–2022			*p*-value
	*n* = 462			*n* = 1,119			
	No. positive samples	Prevalence (%)	95% CI	No. positive samples	Prevalence (%)	95% CI	
Total arbovirus	116	25.1	21.4–29.3	59	5.3	4.1–6.7	0.0001^a^
DENV	67	14.5	11.6–18.0	21	1.9	1.2–2.9	0.0001^a^
DENV-1	28	6.0	4.2–8.6	6	0.5	0.2–1.2	0.0001^a^
DENV-2	33	7.1	5.1–9.9	16	1.4	0.9–2.3	0.0001^a^
DENV-3	6	1.3	0.6–2.9	0	0.0	0.0	0.0010^b^
DENV-4	3	0.6	0.2–2.0	0	0.0	0.0	0.0250^b^
ZIKV	36	7.8	5.7–10.6	3	0.3	0.1–0.8	0.0001^a^
CHIKV	37	8.0	5.9–10.9	37	3.3	2.4–4.5	0.0001^a^
Co-infections	20	4.3	2.8–6.6	3	0.2	0.1–0.8	0.0001^a^

DENV, Dengue virus; ZIKV, Zika virus; CHIKV, Chikungunya virus; 95% CI, 95% confidence interval. Comparisons were made with the ^a^Chi-squared or ^b^Fisher’s exact test (two-tailed).

During the dengue outbreak, the overall prevalence of arboviral RNA was 25.1%, with the highest prevalence for the four dengue serotypes (14.5%), followed by CHIKV (8.0%) and ZIKV (7.8%). In contrast, during the endemic phase, the overall prevalence of arboviral RNA decreased to 5.3% (*p* = 0.0001), with the highest prevalence for CHIKV (3.3%), followed by DENV (1.9%) and ZIKV (0.3%). In addition, co-infections were observed with a frequency of 4.3% during the outbreak and 0.2% during the endemic phase (*p* = 0.0001). These results indicate the simultaneous circulation of DENV, ZIKV, and CHIKV, and the occurrence of co-infections in blood donors during the dengue outbreak and endemic period.

### 3.3 Various arbovirus co-infections were detected in blood donors

**Figure 2:**
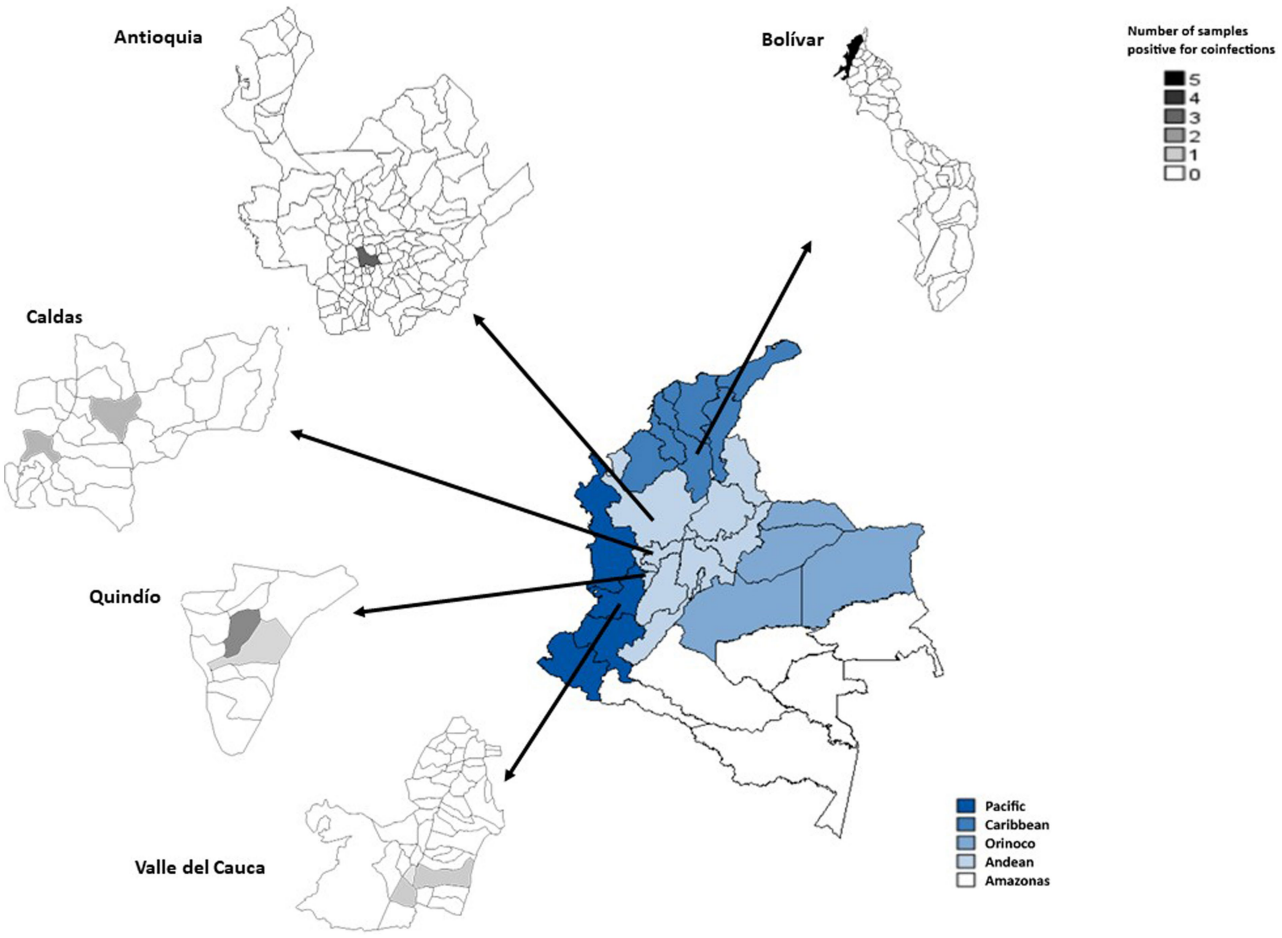
Regions of Colombia, highlighting the provinces and municipalities in which arboviral co-infections were observed during the dengue outbreak (2019–2020). The gradient colors on the map represent the number of samples positive for co-infection.

During the dengue outbreak, more than one arbovirus was detected in 20 of 462 samples. Interestingly, in most cases, two arboviruses were present (14/20), but three or four arboviruses in the same sample were also detected (5/20 and 1/20, respectively; Table 3). Among the most frequent co-infecting arboviruses detected were ZIKV and CHIKV (4/20), followed by DENV-1 and ZIKV (3/20) and DENV-1 and CHIKV (3/20; Table 3). Figure 2 shows the geographical origin of the co-infections observed during the dengue outbreak in Colombia.

**Table 3:** Arbovirus co-infections in blood donors in Colombia during a dengue outbreak (2019–2020).

Co-infections (*n*)	Dengue outbreak 2019–2020				
	*n* = 462				
	Armenia	Cali	Cartagena	Medellín	Manizales
DENV-1 + CHIKV	1	0	0	1	1
DENV-2 + ZIKV	1	1	0	0	0
DENV-1 + DENV-2	0	1	0	0	0
DENV-1 + ZIKV	0	2	0	0	1
DENV-2 + CHIKV	0	0	1	0	0
ZIKV + CHIKV	0	0	1	3	0
DENV-2 + ZIKV + CHIKV	1	0	0	0	0
DENV-1 + DENV-3 + CHIKV	0	1	0	0	0
DENV-3 + ZIKV + CHIKV	0	0	2	0	0
DENV-1 + ZIKV + CHIKV	1	0	0	0	0
DENV-1 + DENV-2 + ZIKV + CHIKV	0	0	1	0	0
Total	4	5	5	4	2

During the endemic phase, the frequency of arbovirus co-infections significantly decreased (three out of 1,119 samples) compared with the dengue outbreak (*p* = 0.0001; Table 2). Two out of three samples came from Medellín, Antioquia (DENV-1 + DENV-2 and ZIKV + CHIKV co-infections); and one case from Manizales, Caldas (DENV-1 + CHIKV co-infection). Thus, in an endemic country, multiple arboviruses can circulate in a single blood donor regardless of the epidemiological period of arbovirus circulation.

### 3.4 The highest reported prevalence of arbovirus in blood donors was observed in endemic areas during the dengue outbreak

As previously mentioned, each city in which samples were collected in two different epidemiological settings was classified as endemic or nonendemic for arbovirus circulation: two cities (Bogotá and Manizales) were classified as nonendemic, whereas four cities (Armenia, Cali, Cartagena, and Medellín) were classified as endemic. We then compared the prevalence of arbovirus circulation in blood donors from each group of cities during the dengue outbreak and the endemic period.

In endemic areas, the highest prevalence of arboviruses was observed in blood donors during the dengue outbreak (30.3%), with infection by any of the four DENV serotypes being the most prevalent (16.8%), followed by infection with CHIKV (12.1%), ZIKV (9.4%), and co-infections (6.7%). These frequencies in endemic areas decreased significantly during the endemic phase, with a global prevalence of arboviruses of 5.5%, caused first by CHIKV (3.5%), followed by DENV (2.0%), of which only serotypes 1 and 2 were observed. A prevalence of 0.3% of co-infections and 0.1% of ZIKV infection was detected (Table 4).

**Table 4:** Prevalence of arboviral RNA in blood donors in dengue endemic and nonendemic areas of Colombia during a dengue outbreak (2019–2020) and an endemic phase (2021–2022).

Endemic areas (Armenia, Cali, Medellín, and Cartagena) (Altitude below 1,984 m.a.s.l.)							
Variable	Dengue outbreak 2019–2020			Dengue endemic phase 2021–2022			*p*-value
	*n* = 297			*n* = 695			
	No. positive samples	Prevalence (%)	95% CI	No. positive samples	Prevalence (%)	95% CI	
Total arbovirus	90	30.3	25.3–35.8	38	5.5	4.0–7.4	0.0001^a^
DENV	50	16.8	13.0–21.5	14	2.0	1.2–3.4	0.0001^a^
DENV-1	24	8.1	5.5–11.8	4	0.6	0.2–1.5	0.0001^a^
DENV-2	24	8.1	5.5–11.8	11	1.6	0.9–2.8	0.0001^a^
DENV-3	4	1.3	0.5–3.5	0	0.0	0.0	0.0090^b^
DENV-4	1	0.3	0.0–2.4	0	0.0	0.0	n.s.^b^
ZIKV	28	9.4	6.6–13.3	1	0.1	0–1	0.0001^a^
CHIKV	36	12.1	8.9–16.4	24	3.5	2.3–5.1	0.0001^a^
Co-infections	20	6.7	4.4–10.2	2	0.3	0.1–1.1	0.0001^a^
Nonendemic areas (Bogotá, Manizales) (Altitude above 1,984 m.a.s.l.)							
Variable	Dengue outbreak 2019–2020			Dengue endemic phase 2021–2022			*p*-value
	*n* = 165			*n* = 424			
	No. positive samples	Prevalence (%)	95% CI	No. positive samples	Prevalence (%)	95% CI	
Total arbovirus	26	15.8^*^	11.0–22.1	21	5.0	3.3–7.5	0.0001^a^
DENV	17	10.3^†^	6.5–16.0	7	1.7	0.8–3.4	0.0001^a^
DENV-1	4	2.4^*^	0.9–6.3	2	0.5	0.1–1.9	n.s.^a^
DENV-2	9	5.5	2.9–10.2	5	1.2	0.5–2.8	0.0400^a^
DENV-3	2	1.2	0.3–4.7	0	0.0	0.0	n.s.^b^
DENV-4	2	1.2	0.3–4.7	0	0.0	0.0	0.0140^b^
ZIKV	8	4.8^†^	2.4–9.4	2	0.5	0.1–1.9	0.0100^a^
CHIKV	1	0.6^*^	0.1–4.2	13	3.1	1.8–5.2	n.s.^a^
Co-infections	0	0.0^*^	0.0	1	0.2	0.0–1.7	n.s.^b^

DENV, Dengue virus; ZIKV, Zika virus; CHIKV, Chikungunya virus; 95% CI, 95% confidence interval. Comparisons were made with ^a^Chi-squared or ^b^Fisher’s exact test. ^†^*p* < 0.05; ^*^p < 0.001; between endemic and nonendemic areas during the dengue outbreak (2019–2020). n.s., not significant.

Additionally, comparing the endemic areas during the dengue outbreak with the nonendemic areas, a significantly lower global prevalence of arboviruses in blood donors was observed in the latter (15.8%), with infections caused by dengue being the first. The prevalence of circulation for the four DENV serotypes (10.3%) was followed by ZIKV (4.8%) and CHIKV (0.6%), and no co-infections were observed (Table 4). The prevalence of arboviruses during the endemic phase was similar in both endemic and nonendemic areas, although only serotypes 1 and 2 were observed for DENV.

### 3.5 Most arbovirus infections in blood donors were asymptomatic or mildly symptomatic

Blood donors who tested positive for viral RNA were contacted to record their sociodemographic, clinical, and epidemiological information. The response rate was 47% (83 of 175 donors), and the results are presented in Table 5. In the nonendemic region, 67% of the infected donors had no travel history to endemic areas. In total, 57% of the survey respondents were asymptomatic, and 43% reported any of the most common arboviral symptoms. The most frequent self-reported symptoms were headache, general malaise, and arthralgia, but none of them were diagnosed with an arboviral disease. No significant differences were observed between endemic and nonendemic areas or between viruses.

**Table 5:** Clinical and epidemiological characteristics of blood donors infected with arbovirus in Colombia.

Characteristic		Areas		Total (*n* = 83; 100%)
		Nonendemic areas (*n* = 18; 22%)	Endemic areas (*n* = 65; 78%)	
Sex	Female	10; 56%	33; 51%	43; 52%
	Male	8; 44%	32; 49%	40; 48%
Donor type	First time	6; 33%	17; 26%	23; 28%
	Non-repetitive	5; 28%	29; 45%	34; 41%
	Repetitive	7; 39%	19; 29%	26; 31%
History of travel to endemic region in the last month	No	12, 67%	37; 57%	49; 59%
	Yes	6; 33%	28; 43%	34; 41%
Arbovirus infection	Dengue	10; 56%	26; 40%	36; 43%
	Zika	2; 11%	9; 14%	11; 13%
	Chikungunya	6; 33%	16; 24%	22; 27%
	Co-infection	0; 0%	14; 22%	14; 17%
Symptomatic	No	13; 72%	34; 52%	47; 57%
	Yes	5; 28%	31; 48%	36; 43%
Headache		5; 28%	21; 32%	26; 31%
Malaise		2; 11%	9; 14%	11; 13%
Arthralgia		0; 0%	8; 12%	8; 10%
Fever/chills		1; 6%	6; 9%	7; 8%
Ocular or retro-orbital pain		1; 6%	6; 9%	7: 8%
Bone or muscle pain		0; 0%	4; 6%	4; 5%
Conjunctivitis		0; 0%	4; 6%	4; 5%
Exanthema		1; 6%	2; 3%	3; 4%
Abdominal pain		0; 0%	3; 5%	3; 4%

Data are presented as percentages. No statistically significant differences were observed between endemic and nonendemic cities (*p* > 0.05).

## 4 Discussion

In this study, we used a hemi-nested RT-PCR approach previously developed in our laboratory (43) to detect arboviral RNA of DENV, ZIKV, and CHIKV in sera from accepted blood donors in Colombia during two different epidemiological periods, one during a dengue outbreak (2019–2020) and the other during a dengue endemic phase (2021–2022). Interestingly, we found a DENV prevalence of 14.5% during the dengue outbreak and 1.9% during the dengue endemic phase, which are significantly higher than the prevalence previously reported in the Americas (<1%) (44–48), the Asian continent (<1%) (49, 50), Saudi Arabia (5.5%) (25), and Portugal (2.3%) (51). Indeed, studies performed in endemic countries during dengue outbreaks did not find a rate of arbovirus viremic donations as high as we found in Colombia during a dengue outbreak (52). However, it should be noted that several studies reporting a low prevalence of DENV RNA in blood donors used samples frozen at −80°C for a long time (46, 53–55), and as it is known viral RNA in serum can degrade over time if stored at temperatures above −70°C (56, 57). The high prevalence observed in this study can be partly explained by the short time between sample collection and processing (maximum 7 days), which enhances RNA stability. On the other hand, enhanced detection of arboviral RNA can also be attributed to the higher analytical sensitivity of the nested PCR approach compared with conventional PCR and real-time PCR (58–61). It is also crucial to consider that a major problem exacerbated by the highly sensitive nature of the hnRT-PCR is the risk of contamination. However, precautions such as the use of dedicated locations and equipment were implemented to minimize this eventuality.

The 2019 dengue outbreak was most severe in Colombia, with 127,553 symptomatic dengue cases reported (33). The high prevalence of DENV in blood donors at this time could be due to the approximately 65% of DENV-infected people who are asymptomatic (62). Consequently, this study shows that the hyperendemic status of the country could also be reflected in the population of accepted blood donors who were probably asymptomatic or presymptomatic at the time of blood donation. In addition to a high prevalence of DENV, other arboviruses such as CHIKV and ZIKV occurred at frequencies of 8% and 7.8% in blood donors, respectively, during this dengue outbreak. Concurrent circulation of four serotypes of DENV, ZIKV, and CHIKV has been reported in the general population of Colombia (36, 63, 64). However, the number of reported symptomatic cases of ZIKV and CHIKV was low in the country during the dengue outbreak and the endemic period (65–71). According to the national epidemiological surveillance system (SIVIGILA), more than 38% of symptomatic dengue cases reported during the dengue outbreak and 30% during the endemic phase were not confirmed by laboratory techniques but registered as probable or as cases with epidemiological links (33, 62). Considering that the clinical symptoms of DENV, ZIKV, and CHIKV infections are nonspecific and include general malaise, headache, and arthralgias/myalgias (64), it is plausible that a proportion of probable dengue cases unconfirmed by laboratory tests include infections with CHIKV and/or ZIKV. However, more studies are needed to confirm this hypothesis.

In hyperendemic countries for arboviruses such as Colombia, co-infections are often found in patients with febrile syndromes compatible with DENV infection, where arboviral RNA from more than one arbovirus is detected (64, 72). Thus, we found co-infections with DENV/ZIKV, DENV/CHIKV, and ZIKV/CHIKV, not to mention co-infections with the three arboviruses. In the context of blood safety, if arboviral RNA in the serum of blood donors is accompanied by infectious viral particles in the blood components obtained from them, co-infections in blood donors could transmit not only one but also several arboviruses to a susceptible recipient. Future studies should measure the quantity, stability, and viability of viral particles in blood components obtained from infected donors.

Donors in whom arboviral RNA was detected were inquired about their sociodemographic, clinical, and epidemiological characteristics within 15 days after blood donation. As expected, most patients reported being asymptomatic after donating blood (57%). Among those who reported symptoms, these were mild and nonspecific, such as headache, general malaise, and arthralgia. Arboviral infections are frequently subclinical or inapparent and therefore insufficient for clinical consultation, even with positive viral or antibody tests (4). Therefore, an infected but asymptomatic subject could donate blood. Blood donors should be educated so that they can notify the blood bank if they notice any symptoms after donating blood. On the other hand, 67% of the blood donors positive for arbovirus who were interviewed and who came from nonendemic areas for dengue stated that they had not traveled to endemic areas in the past 3 months before donating blood. To classify the country’s regions as endemic or nonendemic, 1,984 m.a.s.l. was defined as the cut-off because dengue-positive *Aedes aegypti* vector has been documented in Colombia even at this altitude (42). However, uninfected specimens of this vector have also been observed up to 2,302 m.a.s.l. in Colombia (42) and 2,900 m.a.s.l. in other countries (73). More recently, it was also reported that another vector species, *Aedes albopictus*, can transmit CHIKV at a temperature of 20°C and at high altitude (74). Therefore, epidemiological surveillance studies are required to determine whether infected mosquitoes are circulating at increasingly higher altitudes.

One limitation of this study is that only arboviral RNA from DENV, CHIKV, and ZIKV was detected in individual serum samples from accepted blood donors, which may have contained viral particles that could infect a transfusion recipient. It is important to note that DENV has been isolated in cell cultures (using C6/36 lines) from positive blood donor samples (16). It is also important to consider whether these arboviruses are stable and remain infectious when stored in blood components such as red blood cells, platelets, and plasma. To our knowledge, only one report confirmed that DENV-2 is stable in platelets and red blood cells stored under standard blood bank conditions (75). Additionally, despite the high prevalence of arbovirus in blood donors, there are no reports in Colombia suggesting its transmission through transfusion. Therefore, active hemovigilance studies should be performed to detect arboviruses from index cases of adverse transfusion reactions that present with fever, for example.

In conclusion, for the first time, we report the presence of arboviral RNA of DENV, ZIKV, and CHIKV, as well as co-infections, in sera from blood donors from endemic and nonendemic cities in Colombia. Furthermore, we found that most donors who tested positive had asymptomatic infection or mild and nonspecific symptoms. Finally, these results inform future studies on the risks these viruses can pose to blood safety in Colombia.

## Data availability statement

The raw data supporting the conclusions of this article will be made available by the authors, without undue reservation.

## Ethics statement

The studies involving humans were approved by the Universidad El Bosque Institutional Ethics Committee. The studies were conducted in accordance with the local legislation and institutional requirements. The participants provided their written informed consent to participate in this study.

## Author contributions

BC: Conceptualization, Formal analysis, Methodology, Writing – original draft, Writing – review & editing. AU: Conceptualization, Data curation, Formal analysis, Writing – original draft, Writing – review & editing. TO: Methodology, Writing – review & editing. AR: Funding acquisition, Methodology, Writing – review & editing. OF: Funding acquisition, Methodology, Writing – review & editing. LO: Funding acquisition, Methodology, Writing – review & editing. IF: Funding acquisition, Methodology, Writing – review & editing. DU: Funding acquisition, Methodology, Writing – review & editing. CA: Funding acquisition, Methodology, Writing – review & editing. EC: Conceptualization, Methodology, Writing – review & editing. FD: Conceptualization, Formal analysis, Funding acquisition, Writing – original draft, Writing – review & editing. JC: Conceptualization, Funding acquisition, Writing – review & editing.
